# Genomic architecture of bipolar disorder in Japan: Insights from genomic structural equation modeling

**DOI:** 10.1111/pcn.13906

**Published:** 2025-10-04

**Authors:** Hiroki Kimura, Yukako Nagasaki, Sawako Furukawa, Shiori Ogawa, Takeo Saito, Chikashi Terao, Nakao Iwata, Masashi Ikeda

**Affiliations:** ^1^ Department of Psychiatry Nagoya University Graduate School of Medicine Nagoya Japan; ^2^ Research Center of Health, Physical Fitness and Sports Nagoya University Nagoya Japan; ^3^ Department of Psychiatry Fujita Health University School of Medicine Toyoake Japan; ^4^ Laboratory for Statistical and Translational Genetics RIKEN Center for Integrative Medical Sciences Yokohama Japan; ^5^ Clinical Research Center, Shizuoka General Hospital Shizuoka Japan; ^6^ The Department of Applied Genetics The School of Pharmaceutical Sciences, University of Shizuoka Shizuoka Japan

Bipolar disorder (BD) is a genetically and clinically heterogeneous condition that shares part of its polygenic architecture with schizophrenia (SCZ) and depression.^1^ We previously found that while European BD subtype I (BD1) shows a stronger genetic correlation with SCZ, Japanese BD1 is more strongly correlated with major depressive disorder (MDD),^2^ suggesting that the genetic architecture of BD differs among populations. However, genetic correlations only quantify the association between traits; they do not assess causality or multivariate genetic architecture. By contrast, genomic structural equation modeling (GSEM) models the multivariate genetic architecture of a constellation of traits,^3^ and recent GSEM applications have identified common underlying factors across psychiatric disorders (e.g. a *psychotic dimension*, an *internalizing dimension*) that help explain the shared genetic architecture among these conditions.^4^

Therefore, to examine the multivariate genetic architecture of BD in the Japanese population, we performed GSEM using genome‐wide association study (GWAS) summary statistics for SCZ, BD, and MDD from both European and East Asian populations (nine traits in total). These included updated public data as well as the Japanese BD cohort identical to that reported in our previous study (Table S1).^2^ Detailed methods and results are provided in the Supplementary Information.

First, we estimated pairwise genetic correlations among these nine psychiatric traits using linkage disequilibrium score regression (LDSC) within the GSEM framework. For cross‐population comparisons, we also used Popcorn, a method specifically designed to estimate trans‐ancestry genetic correlations.^5^ Despite using GSEM rather than standalone LDSC, the results largely mirrored our previous findings.^2^ As previously reported, Japanese BD1 showed a stronger genetic correlation with MDD (rg = 0.40) than with SCZ (rg = 0.29), while BD2 exhibited substantial correlations with both SCZ (rg = 0.50) and MDD (rg = 0.77), suggesting genetic heterogeneity (Fig. 1a).

**Fig. 1:**
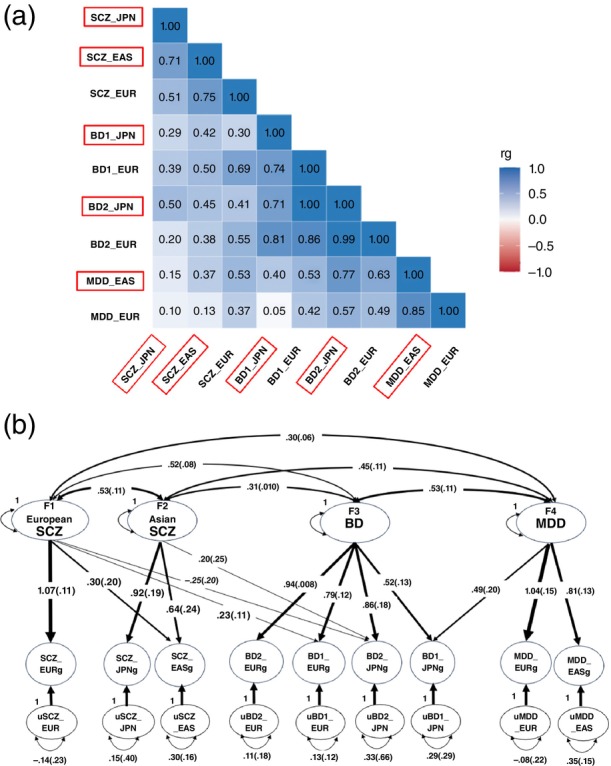
Multivariate genetic architecture of psychiatric disorders in East Asians and Europeans. (a) Genetic correlation of the transpopulation and/or transdisease analyses. The red outlines indicate traits from East Asian samples. Numerical values represent estimates of genetic correlation (rg) (Table S2). (b) GSEM was applied to model the genetic architecture among nine psychiatric traits. The best‐fitting model identified four latent factors: F1 (European SCZ), F2 (Asian SCZ), F3 (BD), and F4 (MDD). Ellipses denote latent variables—both the higher‐order factors (F1–F4) and the phenotype‐specific latent genetic liabilities (e.g. SCZ_EURg, BD1_JPNg) estimated from GWAS summary statistics. Single‐headed arrows indicate standardized factor loadings (the line width reflects the magnitude), double‐headed arrows indicate genetic correlations (covariances) among latent factors, and small curved self‐arrows indicate residual variances. *Slightly negative residual variances (*e.g. *SCZ_EUR, MDD_EUR) were not statistically significant*. Values represent standardized estimates with their Ses in parentheses. Both positive and negative loadings are displayed (negative loadings in italics). BD2_JPN loaded negatively on F1 (−0.25, SE = 0.20; *P* = 0.079) and was retained for model‐fit and conceptual reasons despite not reaching conventional significance. Overall model fit was as follows: CFI = 0.997, SRMR = 0.068, AIC = 81.3. Notation: subscript g denotes a latent genetic liability on the genetic scale (e.g. SCZ_EURg); prefix u denotes a trait‐specific residual genetic factor (e.g. uSCZ_EUR). For readability, the subscript g is omitted for the higher‐order factors F1–F4. AIC, Akaike information criterion; BD, bipolar disorder; CFI, comparative fit index; EAS, East Asian population; EUR, European population; GSEM, genomic structural equation modeling; GWAS, genome‐wide association study; JPN, Japanese population; MDD, major depressive disorder; SCZ, schizophrenia; SE, standard error; SRMR, standardized root mean square residual.

Second, to characterize the multivariate structure underlying these correlations more accurately, we conducted a GSEM factor analysis (Fig. 1b). This analysis identified four latent factors in the best‐fitting model (see Supplementary Result): F1 (European SCZ), F2 (Asian SCZ), F3 (BD), and F4 (MDD). While European BD1 (BD1_EUR) loaded on the same factor as European SCZ (SCZ‐EUR), Japanese BD1 was grouped with MDD phenotypes under F4 (MDD_EAS and MDD_EUR). By contrast, Japanese BD2 grouped together with Asian schizophrenia phenotypes (SCZ_EAS and JPN‐SCZ) under F2, highlighting its closer genetic relationship to psychotic disorders. This pattern suggests that Japanese BD2 shares a similar genetic structure with European BD1. Meanwhile, European BD2 (BD2_EUR) was associated with only the BD factor (F3), indicating a more purely mood‐related genetic profile.

These findings highlight the complex, population‐specific genetic architecture underlying BD, despite several limitations (see Supplementary Limitations). Such differences may partly reflect diagnostic tendencies shaped by Japan's cultural and historical psychiatric context. In Japan, patients with psychotic features are often diagnosed with SCZ rather than BD, and only about 30% of Japanese BD1 cases involve psychotic symptoms—substantially lower than the approximately 50% to 60% in Western cohorts.^2^ These tendencies are consistent with epidemiological data showing a lifetime prevalence of 1% to 2% for BD in Europe (BD1 and BD2 each estimated at ~0.5%–1%),^1^ compared with 0.3% to 0.5% in Japan (BD1 and BD2 each ~0.1%–0.2%).^6^ Additionally, cultural norms emphasizing emotional restraint may contribute to the underrecognition of manic episodes.^7^ If pure mania is regarded as the core symptom of BD, Japanese BD1 may represent a core subgroup within a genetically homogeneous population, characterized by prominent mood symptoms and relatively infrequent psychotic features. These cultural differences could account for our GSEM finding that Japanese BD1 showed loadings on both the BD and MDD factors, reflecting partial genetic overlap. This overlap may help explain variability in treatment response, including potential antidepressant efficacy in some Japanese BD1 cases.

By contrast, BD2_JPN loaded on the SCZ factor, indicating genetic overlap with psychotic disorders. In Japan, psychotic features are not typically emphasized in endogenous depression, which is historically central to MDD.^8^ Consequently, psychotic depression may be classified as SCZ or BD more often in Japan than in Europe, partly reflecting diagnostic tendencies.^9^ These findings also underscore the heterogeneity of BD2 in the Japanese population and suggest that a subset of patients with BD2 may respond more favorably to antipsychotics, especially when psychotic symptoms are prominent. Accordingly, Japanese individuals with BD2—who may present with psychotic symptoms—may benefit from antipsychotic treatment, as supported by previous studies reporting a positive correlation between the proportion of psychotic features and treatment response in acute mania.^10^

In conclusion, our results indicate population differences in the genetic architecture of BD, highlighting the need for tailored diagnostic and treatment strategies.

## Funding Information

This study was supported by grants from the Japan Agency for Medical Research and Development (AMED) under grant numbers JP22wm0425008 (NI), JP23tm0524001 (MI and NI), JP21wm0525024 (MI), 21tm042422 (CT and MI), and JP23dk0307123 (MI and HK); and JSPS KAKENHI grant numbers JP21H02854 (MI), 23K24264 (NI), 24K02381 (MI), and 24K10707 (HK). The funders played no role in the study design, data collection and analysis, decision to publish, or preparation of the manuscript.

## Disclosure statement

NI has received speaker's honoraria from Eisai, Janssen Pharmaceutical KK, Meiji Seika Pharma, Otsuka Pharmaceutical, Sumitomo Pharma, Takeda Pharmaceutical, Mitsubishi Tanabe Pharma, and Viatris. TS has received speaker's honoraria from Otsuka Pharmaceutical and Sumitomo Pharma. MI has received speaker's honoraria from Meiji Seika Pharma, Otsuka Pharmaceutical, Takeda Pharmaceuticals, Lundbeck Japan KK, Mochida Pharmaceutical, Jansen Pharmaceutica KK, Tanabe Mitsubishi Pharma, MSD, Eisai, Kyowa Pharma Chemical, Towa Pharmaceutical, and Viatris.

## Supporting information


**Table S1.** Information on the data sets used in this study.
**Table S2.** Genetic correlations (rg) between psychiatric disorders across East Asian (JPN/EAS) and European (EUR) populations.
**Table S3.** Results of genetic correlations used in the factor analysis.
**Table S4.** Results of exploratory factor analysis (EFA) (3‐ and 5‐factor models).
**Table S5.** Results of the exploratory factor analysis (EFA) (4‐factor model).
**Table S6.** Exploratory factor analysis (EFA) results from the East Asian‐specific genomic structural equation modeling (GSEM).
**Figure S1.** Recruitment sites for bipolar disorder (BD) samples in Japan. (a) Geographical distribution of institutions contributing to the recruitment of participants with BD type I (BD1) and type II (BD2). Red dots indicate the location of each institution. (b) List of institutions and their respective prefectures. The RIKEN sample was obtained through the advanced collaborative study of mood disorders (COSMO) team.

## Data Availability

The data that support the findings of this study are available on request from the corresponding author. The data are not publicly available due to privacy or ethical restrictions.
