# Supplementary material for: Genomic architecture of bipolar disorder in Japan: Insights from genomic structural equation modeling
Source: Psychiatry Clin Neurosci. 2025 Oct 4;79(12):859–60. doi: 10.1111/pcn.13906 (PMC12683609; doi:10.1111/pcn.13906)
Supplement: Supplementary file 1 — Table S1. Information on the data sets used in this study. Table S2. Genetic correlations (rg) between psychiatric disorders across East Asian (JPN/EAS) and European (EUR) populations. Table S3. Results of genetic correlations used in the factor analysis. Table S4. Results of exploratory factor analysis (EFA) (3‐ and 5‐factor models). Table S5. Results of the exploratory factor analysis (EFA) (4‐factor model). Table S6. Exploratory factor analysis (EFA) results from the East Asian‐specific genomic structural equation modeling (GSEM). Figure S1. Recruitment sites for bipolar disorder (BD) samples in Japan. (a) Geographical distribution of institutions contributing to the recruitment of participants with BD type I (BD1) and type II (BD2). Red dots indicate the location of each institution. (b) List of institutions and their respective prefectures. The RIKEN sample was obtained through the advanced collaborative study of mood disorders (COSMO) team. [file PCN-79-859-s001.docx]

**Supporting Information:**

**Supplementary Methods**

**Ethical statement**

For the bipolar disorder (BD) and schizophrenia (SCZ) genome-wide association studies (GWAS) in the Japanese population, written informed consent was obtained from all subjects following a thorough explanation of the study, including that their anonymity would be preserved.^1, 2^ This study, which conformed to the provisions of the Declaration of Helsinki, was approved by the ethics committees of Nagoya University and other participating universities.

**Data sets used in this study**

In this study, we aimed to evaluate the genetic architecture of BD in the Japanese population by using summary statistics from psychiatric disorders in East Asian populations, including Japan, as well as corresponding data sets from European populations. For the European (EUR) samples, we utilized summary statistics from the Psychiatric Genomics Consortium (PGC), including MDD_EUR,^3^ SCZ_EUR,^4^ BD1_EUR,^5^ and BD2_EUR.^5^ These data sets are publicly available at <https://pgc.unc.edu/for-researchers/download-results/>. For the East Asian (EAS) samples, we used Japanese GWAS summary statistics for BD and SCZ,^6^ along with East Asian data sets from the PGC for MDD_EAS^7^ and SCZ_EAS.^8^ We note that SCZ_JPN and SCZ_EAS include partially overlapping samples, with 547 cases and 540 controls from SCZ_JPN also included in SCZ_EAS. Given the large overall sample size of SCZ_EAS (22,778 cases and 35,362 controls), we consider that this minor overlap has a negligible impact on our analyses. Detailed information on these summary statistics can be seen in Table S1 and Figure S1. The Japanese cohort analyzed in this study is the same as that used in our previous publication.^6^ In the present study, however, we applied genomic structural equation modeling (GSEM) using the R package GSEM to estimate trans-diagnostic genetic correlations, which were not assessed in the earlier work.^6^ By leveraging GSEM, we explored trans-diagnostic genetic structures across disorders and populations—a perspective not examined previously. Accordingly, the genetic correlations and latent factor structure reported here are novel findings and offer new insights into the shared and distinct genetic architecture of psychiatric disorders.

**Genomic structural equation modeling**

We applied GSEM to investigate the multivariate genetic architecture of psychiatric disorders.^9^ GSEM software is an R package available from GitHub at: <https://github.com/GenomicSEM/GenomicSEM>. This approach consists of three main stages:

**Stage 1: Preparation of summary statistics**

The first step in running a GSEM model is to munge the summary statistics. The munge function works to convert summary statistics to the format expected by **linkage disequilibrium score regression (LDSC)** (i.e., on a z-statistic metric). The summary statistics used in this study are described in Table S1. Before munging, the sum of the effective sample size is calculated based on a sample size of each study as instructed by the manual.

**Stage 2: Multivariable LDSC**

Next, we conducted multivariable LDSC using the ldsc() function implemented in the GSEM package. LD scores were aligned with the ancestry of the respective summary statistics (e.g., European summary statistics were paired with European LD scores). We employed the Popcorn method to estimate genetic correlations between traits across different ancestries.^10^ The Popcorn package used for this analysis is publicly available at <https://github.com/brielin/Popcorn>. A heatmap was generated in R to visualize the genetic correlations (rg) among nine psychiatric traits (Figure 1a).

For the factor analysis described in Stage 3, we performed LDSC using the European LD score because trans-ancestral comparisons are not directly supported in the GSEM LDSC framework. In this step, we used a modified version of LDSC that populates the off-diagonal elements of the sampling covariance matrix (V) to reflect dependencies between traits due to overlapping samples. This procedure yielded an empirical genetic covariance matrix (S) and a corresponding sampling covariance matrix (V), which served as inputs for subsequent modeling.

**Stage 3: Factor modeling using EFA and CFA**

To investigate the underlying genetic architecture across multiple traits, we first conducted an exploratory factor analysis (EFA) using the genetic covariance matrix based on the S and V matrices obtained in Stage 2. This step allowed us to identify potential latent factors that capture shared genetic variance across phenotypes. Model fit was assessed based on: (i) cumulative variance explained, (ii) sum of square (SS) loadings (with a threshold of ≥1.0), and (iii) the proportion of variance explained by each factor (≥10%). Traits with absolute factor loadings ≥ 0.20 were selected for inclusion in the CFA.

Based on the EFA results and theoretical considerations, we then specified CFA models to test the fit of the proposed latent structure formally. The CFA model incorporated both latent genetic factors and observed genetic components of individual traits. Parameters were estimated by minimizing the discrepancy between the model-implied covariance matrix (Σ) and the empirical matrix (S). CFA was performed using weighted least squares estimation, which accounts for variation in the precision of genetic covariance estimates. Model fit was evaluated using conventional indices, including the comparative fit index (CFI) and the standardized root mean square residual (SRMR). In our CFA, we adopted conventional thresholds to evaluate model fit, following established guidelines^9^: **CFI ≥ 0.90** was considered acceptable, with **≥ 0.95** indicating good fit; **SRMR ≤ 0.08** was considered acceptable; the **Akaike information criterion (AIC)** was used for relative model comparison, with lower values indicating better fit. Following genomic SEM conventions, phenotype-specific genetic liabilities were modeled as single-indicator latent variables and plotted as ellipses. Some standardized factor loadings exceeded 1.0 because of the high saturation of traits and correlated latent factors. This is a known and acceptable phenomenon in CFA and does not indicate model misfit.^9^

**Supplementary results**

**Genetic correlations analysis**

Multivariable LD score regression (LDSC) was performed using the GSEM package. The strong correlations observed between BD1_JPN and BD2_JPN (rg = 0.71), as well as between BD1_EUR and BD2_EUR (rg = 0.86), support the hypothesis that BD1 and BD2 lie on a shared genetic spectrum, although the relative strength of their correlations with SCZ and MDD may vary across ancestries. Notably, Japanese BD1 showed a stronger genetic correlation with MDD (e.g., MDD_EAS, rg = 0.40) than with SCZ (e.g., SCZ_JPN, rg = 0.29), whereas European BD1 was more strongly correlated with European SCZ (rg = 0.69) than with European MDD (rg = 0.42). Interestingly, Japanese BD2 exhibited high genetic correlations with both SCZ (SCZ_JPN rg= 0.50; SCZ_EAS rg = 0.45) and MDD (MDD_EAS rg = 0.77), suggesting that BD2 in Japan may represent a genetically heterogeneous subtype that bridges affective and psychotic dimensions. The LDSC and Popcorn results are described in more detail in Table S2.

**GSEM analysis**

EFA was performed based on the results of the multivariable LDSC with the European LD score by the GSEM package because trans-ancestral comparisons are not directly supported in the GSEM LDSC framework. The LDSC results using the European LD score are presented in Table S3.

Initially, w**e conducted exploratory factor analyses (EFA) with 3-, 4- and 5-factor solutions (Table S4, S5).** In the 3-factor model (Table S4), notable patterns were observed, such as MDD and BD1_JPN loading on the same factor and BD2 clustering with SCZ_EAS. Nevertheless, in the CFA, the 3-factor model demonstrated poor model fit (e.g., CFI = 0.98, SRMR = 0.129, AIC = 127), performing less favorably compared to the 4-factor model. **The 5-factor model (Table S4) explained a greater proportion of variance; however, it failed to converge in CFA. As a result, model fit indices could not be estimated and the CFA output was null. These issues suggest that the 5-factor solution may suffer from overextraction and lacks psychometric validity in this dataset.**

By contrast, a 4-factor model, while not the absolute best-fitting solution, provided the most acceptable balance of model fit and conceptual interpretability. This solution comprised

F1 (European SCZ), F2 (Asian SCZ), F3 (BD), and F4 (MDD), with a cumulative variance of 0.892 and each accounting for between 13.5% and 31.0% of the overall variance explained (F1 of 0.135, F2 of 0.198, F3 of 0.31, and F4 of 0.249). Each of the four factors had high SS loadings (F1 SS of 1.21, F2 SS of 1.78, F3 SS of 2.79, and F4 SS of 2.24). The results of the 4-factor EFA are summarized in Table S5.

The subsequent CFA based on this 4-factor EFA provided an excellent model fit (e.g., CFI = 0.997; SRMR = 0.068; AIC = 81.3) and is shown in Figure 1b. Regarding individual paths, the loading of BD2_EUR on F3 (Asian SCZ) was negligible and nonsignificant (p = 0.73) and was therefore excluded from the final model. By contrast, although the loading of BD2_JPN on F1 (European SCZ) was negative and did not reach conventional significance (p = 0.079), it was retained because of its contribution to model fit and conceptual clarity. Traits loading most strongly on F3 included BD2_EUR (0.94 ± 0.08), BD1_EUR (0.79 ± 0.12), BD2_JPN (0.86 ± 0.18), and BD1_JPN (0.52 ± 0.13). Traits loading most strongly on F4 included MDD_EUR (1.04 ± 0.15) and MDD_EAS (0.81 ± 0.13). SCZ traits loaded most strongly on F1 and F2 included SCZ_EUR (1.07 ± 0.11) and on F2 included SC_JPN (0.92 ± 0.19) and SCZ_EAS (0.64 ± 0.24). Moderate correlations were found between F1 and F2 (*r* = 0.53), F1 and F3 (*r* = 0.52), F2 and F4 (*r* = 0.45), and F3 and F4 (*r* = 0.53). While European BD1 (BD1_EUR) loaded on the same factor as SCZ_EUR (0.23 ± 0.11), BD1_JPN was grouped with MDD phenotypes (0.49 ± 0.20) under F4 (MDD_EAS and MDD_EUR). By contrast, Japanese BD2 (BD2_JPN) clustered with Asian SCZ phenotypes under F2 (SCZ_EAS and SCZ_JPN), emphasizing its genetic proximity to psychotic disorders. However, European BD2 (BD2_EUR) loaded only onto the BD factor (F3), suggesting a more distinct mood-related profile. To ensure better interpretability and reflect prior EFA findings, the factor loading of BD2_JPN on F2 was fixed at 0.20 to represent a weak but hypothesized non-zero association with the psychotic factor. In addition, the loading of SCZ_EAS on F2 was fixed at 0.30, reflecting its strong theoretical and empirical contribution to the psychotic dimension observed in the EFA. These constraints were introduced to reduce estimation instability and improve model convergence.

**Supplementary Limitations**

This study has several limitations. First, large disparities in sample size and statistical power between East Asian and European GWAS may have reduced the stability and precision of genetic covariance estimates, potentially affecting the robustness of the GSEM results. Second, although GSEM does not explicitly model trans-ethnic architecture, results using European LD scores were largely consistent with our trans-ethnic correlations, supporting interpretability. We initially estimated genetic correlations (rg) using European LD scores to ensure stability and comparability across traits. To specifically evaluate trans-ethnic similarity, we additionally applied popcorn to estimate cross-ancestry rg, which are presented in Fig. 1a. ^10^ Therefore, to examine this point further, we conducted a genomic SEM analysis using only ancestry-matched EAS LD scores and East Asian summary statistics. EFA identified a 2-factor solution (Table S6). The CFA model demonstrated excellent fit (χ² = 1.93, df = 3, p = 0.59; CFI = 1.000; SRMR = 0.056). Standardized loadings supported the EFA-derived structure, with SCZ_JPN (0.72) and SCZ_EAS (0.99) loading on the SCZ factor, and BD1_JPN (0.67) and MDD_EAS (0.60) on the mood factor. BD2_JPN showed cross-loadings (–0.36 on Factor1, 1.39 on Factor 2), suggesting an intermediate genetic profile between the mood and psychosis spectrums. However, this EAS-only model did not fully recapitulate the shared liability of BD2 with SCZ observed in the trans-ancestry model. The attenuation and imbalance of BD2’s cross-loadings imply that ancestry-specific GSEM, while informative, may not adequately reflect the trans-ancestry complexity and pleiotropic nature of BD2. Taken together, these findings highlight the potential utility of European LD scores when modeling broader cross-population genetic architectures. Moreover, future methodological advances enabling trans-ancestry GSEM will be essential to comprehensively model cross-population genetic architectures and to further clarify both shared and population-specific latent structures.

**Table S1.** Information on the data sets used in this study.

| **Traits** | **Summary statistics** | **PGC_Database** | **Sample number (case/control)** |
| --- | --- | --- | --- |
| SCZ_EUR | Nature. 2022;604(7906):502-8 | scz2022 | 76,755/24,3649 |
| BD1_EUR | Nat Genet. 2021;53(6):817-29 | bip2021 | 25,060/50,981 |
| BD2_EUR | Nat Genet. 2021;53(6):817-29 | bip2021 | 6,781/50,981 |
| MDD_EUR | Nat Neurosci. 2019;22(3):343-52 | mdd2019edinburgh | 246,363/561,190 |
| SCZ_EAS | Nat Genet. 2019;51(12):1670-8 | scz2019asi | 22,778/35,362 |
| MDD_EAS | Nat Genet. 2024;56(2):222-33 | mdd2023diverse | 88,316/902,757 |
| SCZ_JPN | Psychiatry Clin Neurosci. 2023;77(2):118-9 | - | 1,941/7,408 |
| BD1_JPN | Psychiatry Clin Neurosci. 2023;77(2):118-9 | - | 1480/27,146 |
| BD2_JPN | Psychiatry Clin Neurosci. 2023;77(2):118-9 | - | 1,380/27,145 |

Note: PGC; Psychiatric Genomics Consortium, SCZ; schizophrenia, BD: bipolar disorder, MDD; major depressive disorder. EUR; European population, EAS; East Asian population.

**Table S2.** **Genetic correlations (rg) between psychiatric disorders across East Asian (JPN/EAS) and European (EUR) populations.**

| **Trait1** | **Trait2** | **rg** | **SE** | **p-value** | |
| --- | --- | --- | --- | --- | --- |
|  |  |  |  | **rg > 0** | **rg < 1** |
| **SCZ_JPN** | SCZ_EAS | 0.71 | 0.07 | 1.8E-24 | 1.7E-05 |
| **SCZ_JPN** | BD1_JPN | 0.29 | 0.13 | 1.3E-02 | 2.4E-08 |
| **SCZ_JPN** | BD2_JPN | 0.50 | 0.19 | 4.2E-03 | 4.2E-03 |
| **SCZ_JPN** | MDD_EAS | 0.15 | 0.13 | 1.2E-01 | 3.1E-11 |
| **SCZ_JPN** | SCZ_EUR | 0.51 | 0.12 | 6.9E-06 | 2.5E-05 |
| **SCZ_JPN** | BD1_EUR | 0.39 | 0.08 | 9.8E-07 | 1.6E-13 |
| **SCZ_JPN** | BD2_EUR | 0.20 | 0.15 | 4.8E-02 | 2.0E-07 |
| **SCZ_JPN** | MDD_EUR | 0.10 | 0.08 | 1.1E-01 | 2.6E-28 |
| **SCZ_EAS** | BD1_JPN | 0.42 | 0.08 | 7.6E-08 | 2.1E-13 |
| **SCZ_EAS** | BD2_JPN | 0.45 | 0.11 | 2.1E-05 | 2.9E-07 |
| **SCZ_EAS** | MDD_EAS | 0.37 | 0.08 | 1.9E-06 | 1.7E-15 |
| **SCZ_EAS** | SCZ_EUR | 0.75 | 0.12 | 2.9E-11 | 1.8E-02 |
| **SCZ_EAS** | BD1_EUR | 0.50 | 0.06 | 1.6E-16 | 1.8E-16 |
| **SCZ_EAS** | BD2_EUR | 0.38 | 0.10 | 6.9E-05 | 4.3E-10 |
| **SCZ_EAS** | MDD_EUR | 0.13 | 0.05 | 3.9E-03 | 3.0E-76 |
| **BD1_JPN** | BD2_JPN | 0.71 | 0.24 | 1.5E-03 | 1.1E-01 |
| **BD1_JPN** | MDD_EAS | 0.40 | 0.16 | 6.2E-03 | 8.8E-05 |
| **BD1_JPN** | SCZ_EUR | 0.30 | 0.12 | 6.7E-03 | 8.9E-09 |
| **BD1_JPN** | BD1_EUR | 0.74 | 0.12 | 1.2E-10 | 1.7E-02 |
| **BD1_JPN** | BD2_EUR | 0.81 | 0.16 | 2.6E-07 | 1.3E-01 |
| **BD1_JPN** | MDD_EUR | 0.05 | 0.10 | 2.9E-01 | 1.1E-21 |
| **BD2_JPN** | MDD_EAS | 0.77 | 0.21 | 1.2E-04 | 1.4E-01 |
| **BD2_JPN** | SCZ_EUR | 0.41 | 0.22 | 3.1E-02 | 3.9E-03 |
| **BD2_JPN** | BD1_EUR | 1.00 | 0.47 | 1.6E-02 | 5.0E-01 |
| **BD2_JPN** | BD2_EUR | 0.99 | 0.55 | 3.6E-02 | 4.9E-01 |
| **BD2_JPN** | MDD_EUR | 0.57 | 0.39 | 7.1E-02 | 1.3E-01 |
| **MDD_EAS** | SCZ_EUR | 0.53 | 0.15 | 2.6E-04 | 1.1E-03 |
| **MDD_EAS** | BD1_EUR | 0.52 | 0.13 | 4.0E-05 | 1.1E-04 |
| **MDD_EAS** | BD2_EUR | 0.63 | 0.24 | 4.6E-03 | 6.1E-02 |
| **MDD_EAS** | MDD_EUR | 0.85 | 0.19 | 2.4E-06 | 2.1E-01 |
| **SCZ_EUR** | BD1_EUR | 0.69 | 0.03 | 2.3E-117 | 2.5E-25 |
| **SCZ_EUR** | BD2_EUR | 0.55 | 0.04 | 2.5E-43 | 1.2E-29 |
| **SCZ_EUR** | MDD_EUR | 0.37 | 0.07 | 6.3E-08 | 1.1E-19 |
| **BD1_EUR** | BD2_EUR | 0.86 | 0.06 | 6.8E-47 | 9.8E-03 |
| **BD1_EUR** | MDD_EUR | 0.42 | 0.09 | 1.5E-06 | 5.8E-11 |
| **BD2_EUR** | MDD_EUR | 0.49 | 0.14 | 2.3E-04 | 1.3E-04 |

Note: **rg = genetic correlation; SE = standard error of rg. The left p-value corresponds to a one-tailed test for whether the genetic correlation is significantly greater than zero (rg > 0), as estimated by LDSC, using population-specific LD score files (EAS or EUR). The right p-value corresponds to a one-tailed test for whether the genetic correlation was significantly less than one (rg < 1).** These tests help identify both meaningful positive correlations and evidence of nonidentical genetic architectures across traits. **Cells highlighted in blue indicate values derived from Popcorn estimates. Trait labels: SCZ = schizophrenia, BD1 = bipolar disorder type I, BD2 = bipolar disorder type II, MDD = major depressive disorder; JPN = Japanese population, EAS = East Asian ancestry, EUR = European ancestry.**

**Table S3.** Results of genetic correlations used in the factor analysis.

| **Trait1** | **Trait2** | **rg** | **SE** | **p-value** | |
| --- | --- | --- | --- | --- | --- |
|  |  |  |  | **rg > 0** | **rg < 1** |
| **SCZ_JPN** | SCZ_EAS | 0.72 | 0.074 | 6.5E-23 | 1.3E-04 |
| **SCZ_JPN** | BD1_JPN | 0.42 | 0.13 | 6.2E-04 | 7.2E-06 |
| **SCZ_JPN** | BD2_JPN | 0.65 | 0.23 | 3.2E-03 | 7.3E-02 |
| **SCZ_JPN** | MDD_EAS | 0.14 | 0.15 | 1.8E-01 | 3.5E-08 |
| **SCZ_JPN** | SCZ_EUR | 0.53 | 0.04 | 3.2E-34 | 8.7E-26 |
| **SCZ_JPN** | BD1_EUR | 0.34 | 0.063 | 2.1E-08 | 1.3E-25 |
| **SCZ_JPN** | BD2_EUR | 0.20 | 0.089 | 9.9E-03 | 3.3E-19 |
| **SCZ_JPN** | MDD_EUR | 0.37 | 0.16 | 1.3E-02 | 1.0E-04 |
| **SCZ_EAS** | BD1_JPN | 0.46 | 0.089 | 8.0E-08 | 1.3E-09 |
| **SCZ_EAS** | BD2_JPN | 0.41 | 0.149 | 2.9E-03 | 3.9E-05 |
| **SCZ_EAS** | MDD_EAS | 0.40 | 0.097 | 1.7E-05 | 4.2E-10 |
| **SCZ_EAS** | SCZ_EUR | 0.67 | 0.03 | 1.2E-111 | 5.2E-28 |
| **SCZ_EAS** | BD1_EUR | 0.41 | 0.037 | 1.9E-29 | 3.5E-55 |
| **SCZ_EAS** | BD2_EUR | 0.30 | 0.057 | 5.3E-08 | 1.6E-34 |
| **SCZ_EAS** | MDD_EUR | 0.49 | 0.10 | 2.1E-06 | 1.1E-06 |
| **BD1_JPN** | BD2_JPN | 0.82 | 0.29 | 2.5E-03 | 2.8E-01 |
| **BD1_JPN** | MDD_EAS | 0.65 | 0.22 | 1.6E-03 | 6.3E-02 |
| **BD1_JPN** | SCZ_EUR | 0.39 | 0.056 | 9.4E-13 | 2.9E-27 |
| **BD1_JPN** | BD1_EUR | 0.72 | 0.084 | 4.6E-18 | 4.7E-04 |
| **BD1_JPN** | BD2_EUR | 0.61 | 0.10 | 1.3E-08 | 1.9E-04 |
| **BD1_JPN** | MDD_EUR | 0.65 | 0.23 | 3.1E-03 | 7.2E-02 |
| **BD2_JPN** | MDD_EAS | 0.70 | 0.34 | 2.2E-02 | 2.0E-01 |
| **BD2_JPN** | SCZ_EUR | 0.29 | 0.091 | 6.2E-04 | 4.8E-15 |
| **BD2_JPN** | BD1_EUR | 0.66 | 0.13 | 2.1E-07 | 5.7E-03 |
| **BD2_JPN** | BD2_EUR | 0.74 | 0.19 | 4.1E-05 | 9.3E-02 |
| **BD2_JPN** | MDD_EUR | 0.55 | 0.38 | 7.5E-02 | 1.2E-01 |
| **MDD_EAS** | SCZ_EUR | 0.32 | 0.061 | 4.4E-08 | 3.9E-28 |
| **MDD_EAS** | BD1_EUR | 0.32 | 0.081 | 3.5E-05 | 6.4E-17 |
| **MDD_EAS** | BD2_EUR | 0.44 | 0.13 | 5.1E-04 | 2.7E-05 |
| **MDD_EAS** | MDD_EUR | 1.64 | 0.33 | 5.6E-07 | 9.7E-01 |
| **SCZ_EUR** | BD1_EUR | 0.68 | 0.034 | 3.5E-90 | 7.0E-20 |
| **SCZ_EUR** | BD2_EUR | 0.54 | 0.043 | 1.8E-36 | 2.7E-25 |
| **SCZ_EUR** | MDD_EUR | 0.37 | 0.065 | 7.0E-09 | 8.3E-22 |
| **BD1_EUR** | BD2_EUR | 0.86 | 0.064 | 8.5E-41 | 1.6E-02 |
| **BD1_EUR** | MDD_EUR | 0.41 | 0.092 | 3.4E-06 | 1.9E-10 |
| **BD2_EUR** | MDD_EUR | 0.49 | 0.14 | 2.4E-04 | 1.6E-04 |

Note: **rg** = genetic correlation; **SE** = standard error of rg. The left p-value corresponds to a one-tailed test assessing whether the genetic correlation (rg) is significantly greater than zero (rg > 0), as estimated by LDSC using population-specific LD score files (EUR). The right p-value corresponds to a one-tailed test assessing whether the genetic correlation (rg) is significantly less than one (rg < 1), also estimated by LDSC. These tests help identify both meaningful positive correlations and evidence of nonidentical genetic architectures across traits. We used EUR-based LD scores because the GSEM framework does not explicitly account for trans-ethnic linkage disequilibrium structure. Trait labels: **SCZ** = schizophrenia, **BD1** = bipolar disorder type I, **BD2** = bipolar disorder type II, **MDD** = major depressive disorder; **JPN** = Japanese population, **EAS** = East Asian ancestry, **EUR** = European ancestry.

**Table S4.** Results of EFA (3- and 5-factor models).

| **Trait** | **F1**  **(BD + EUR-SCZ)** | **F2**  **(MDD)** | **F3**  **(EAS-SCZ)** |
| --- | --- | --- | --- |
| **SCZ_JPN** | –0.229 | –0.137 | 1.179 |
| **SCZ_EUR** | 0.566 |  | 0.323 |
| **BD1_JPN** | 0.572 | 0.266 |  |
| **BD1＿EUR** | 1.157 | –0.184 | –0.115 |
| **MDD_EAS** | –0.103 | 1.115 | –0.159 |
| **MDD_EUR** |  | 1.024 |  |
| **BD2_JPN** | 0.36 | 0.181 | 0.412 |
| **BD2_EUR** | 1.003 |  | –0.256 |
| **SCZ_EAS** |  | 0.104 | 0.722 |
| **Metric** | **Factor 1** | **Factor 2** | **Factor 3** |
| **SS loadings** | 3.192 | 2.47 | 2.3 |
| **Proportion Var** | 0.355 | 0.274 | 0.256 |
| **Cumulative Var** | 0.355 | 0.629 | 0.885 |

| **Trait** | **F1**  **(MDD)** | **F2**  **(BD)** | **F3**  **(EAS-SCZ)** | **F4**  **(EUR-SCZ)** | **F5**  **(EUR-BD1)** |
| --- | --- | --- | --- | --- | --- |
| **SCZ_JPN** | –0.105 |  | 1.035 | 0.169 |  |
| **SCZ_EUR** |  | 0.225 |  | 0.903 |  |
| **BD1_JPN** |  |  |  | 1.039 |  |
| **BD1＿EUR** | –0.153 | 0.632 | –0.127 | 0.357 | 0.324 |
| **MDD_EAS** | 1.018 |  | –0.151 |  |  |
| **MDD_EUR** | 0.964 |  |  |  |  |
| **BD2_JPN** |  | 0.551 | 0.544 | –0.329 | 0.162 |
| **BD2_EUR** |  | 1.079 |  | 0.147 | –0.187 |
| **SCZ_EAS** | 0.119 | –0.171 | 0.433 | 0.518 | 0.116 |
| **Metric** | **Factor 1** | **Factor 2** | **Factor 3** | **Factor 4** | **Factor 5** |
| **SS loadings** | 2.028 | 1.957 | 1.611 | 1.375 | 1.28 |
| **Proportion Var** | 0.225 | 0.217 | 0.179 | 0.153 | 0.142 |
| **Cumulative Var** | 0.225 | 0.443 | 0.622 | 0.775 | 0.917 |

Note: Traits were allowed to load on multiple factors, reflecting an overlapping polygenic architecture. SCZ, schizophrenia; BD, bipolar disorder; MDD, major depressive disorder; JPN, Japanese population; EAS, East Asian population; EUR, European population; SS Loadings, sum of squared loadings; Proportion Var, proportion of variance explained; Cumulative Var, cumulative variance explained.

**Table S5.** Results of the exploratory factor analysis (EFA) (4-factor model).

| **Trait** | **F1**  **(EUR-SCZ)** | **F2**  **(EAS-SCZ)** | **F3**  **(BD)** | **F4**  **(MDD)** |
| --- | --- | --- | --- | --- |
| SCZ_JPN | 0.104 | 1.048 |  |  |
| SCZ_EAS | 0.422 | 0.578 | –0.138 | 0.163 |
| BD1_JPN |  | 0.146 | 0.633 | 0.23 |
| BD2_JPN | –0.326 | 0.453 | 0.749 |  |
| MDD_EAS |  | –0.185 |  | 1.075 |
| SCZ_EUR | 0.871 | 0.135 | 0.161 |  |
| BD1_EUR | 0.357 |  | 0.87 | –0.135 |
| BD2_EUR | 0.18 | –0.252 | 1.011 |  |
| MDD_EUR |  |  |  | 0.982 |
| **Metric** | **Factor 1** | **Factor 2** | **Factor 3** | **Factor 4** |
| SS Loadings | 1.219 | 1.783 | 2.79 | 2.245 |
| Proportion Var | 0.135 | 0.198 | 0.31 | 0.249 |
| Cumulative Var | 0.135 | 0.333 | 0.643 | 0.892 |

Note: Traits were allowed to load on multiple factors, reflecting an overlapping polygenic architecture. SCZ, schizophrenia; BD, bipolar disorder; MDD, major depressive disorder; JPN, Japanese population; EAS, East Asian population; EUR, European population; SS Loadings, sum of squared loadings; Proportion Var, proportion of variance explained; Cumulative Var, cumulative variance explained.

**Table S6.** EFA results from the East Asian-specific GSEM.

| Trait | F1 (Mood disorder) | F2 (SCZ) |
| --- | --- | --- |
| SCZ_JPN |  | 1.002 |
| BD1_JPN | 0.706 |  |
| BD2_JPN | 0.935 | 0.137 |
| MDD_EAS | 0.869 | –0.185 |
| SCZ_EAS | 0.11 | 0.67 |
| Metric | Factor 1 | Factor 2 |
| SS loadings | 2.14 | 1.507 |
| Proportion Var | 0.428 | 0.301 |
| Cumulative Var | 0.428 | 0.729 |

Note: Traits were allowed to load on multiple factors, reflecting an overlapping polygenic architecture. SCZ, schizophrenia; BD, bipolar disorder; MDD, major depressive disorder; JPN, Japanese population; EAS, East Asian population; EUR, European population; SS Loadings, sum of squared loadings; Proportion Var, proportion of variance explained; Cumulative Var, cumulative variance explained.

**Figure S1.** Recruitment sites for bipolar disorder (BD) samples in Japan. a) Geographical distribution of institutions contributing to the recruitment of participants with BD type I (BD1) and type II (BD2). Red dots indicate the location of each institution. b) List of institutions and their respective prefectures. The RIKEN sample was obtained through the Advanced Collaborative Study of Mood Disorders (COSMO) team.

**Author contributions:**

HK, NI, and MI contributed to the conception and study design. HK, YN, SF, SO, TS, CT, and NI provided substantial contributions to the analysis and interpretation of the clinical data. HK, YN, and MI wrote the first draft of the article. All authors contributed to and approved the final version of the manuscript.

**References**

1. Ikeda M, Takahashi A, Kamatani Y, et al. A genome-wide association study identifies two novel susceptibility loci and trans population polygenicity associated with bipolar disorder. *Mol Psychiatry*. 2018; **23**: 639-47.

2. Ikeda M, Takahashi A, Kamatani Y, et al. Genome-Wide Association Study Detected Novel Susceptibility Genes for Schizophrenia and Shared Trans-Populations/Diseases Genetic Effect. *Schizophr Bull*. 2019; **45**: 824-34.

3. Howard DM, Adams MJ, Clarke TK, et al. Genome-wide meta-analysis of depression identifies 102 independent variants and highlights the importance of the prefrontal brain regions. *Nat Neurosci*. 2019; **22**: 343-52.

4. Trubetskoy V, Pardinas AF, Qi T, et al. Mapping genomic loci implicates genes and synaptic biology in schizophrenia. *Nature*. 2022; **604**: 502-8.

5. Mullins N, Forstner AJ, O'Connell KS, et al. Genome-wide association study of more than 40,000 bipolar disorder cases provides new insights into the underlying biology. *Nat Genet*. 2021; **53**: 817-29.

6. Saito T, Ikeda M, Terao C, et al. Differential genetic correlations across major psychiatric disorders between Eastern and Western countries. *Psychiatry Clin Neurosci*. 2023; **77**: 118-9.

7. Meng X, Navoly G, Giannakopoulou O, et al. Multi-ancestry genome-wide association study of major depression aids locus discovery, fine mapping, gene prioritization and causal inference. *Nat Genet*. 2024; **56**: 222-33.

8. Lam M, Chen CY, Li Z, et al. Comparative genetic architectures of schizophrenia in East Asian and European populations. *Nat Genet*. 2019; **51**: 1670-8.

9. Grotzinger AD, Rhemtulla M, de Vlaming R, et al. Genomic structural equation modelling provides insights into the multivariate genetic architecture of complex traits. *Nat Hum Behav*. 2019; **3**: 513-25.

10. Brown BC, Asian Genetic Epidemiology Network Type 2 Diabetes C, Ye CJ, Price AL, Zaitlen N. Transethnic Genetic-Correlation Estimates from Summary Statistics. *Am J Hum Genet*. 2016; **99**: 76-88.
